# Parameter Estimation Based on Sigmoid Transform in Wideband Bistatic MIMO Radar System under Impulsive Noise Environment

**DOI:** 10.3390/s19020232

**Published:** 2019-01-09

**Authors:** Li Li, Nicolas H. Younan, Xiaofei Shi

**Affiliations:** 1 College of Information Engineering, Dalian University, Dalian 116622, China; 2 Department of Electrical and Computer Engineering, Mississippi State University, Starkville, MS 39762, USA; shixiaofei@dlmu.edu.cn; 3 Information Science and Technology College, Dalian Maritime University, Dalian 116026, China

**Keywords:** bistatic MIMO radar, alpha stable distribution, Sigmoid wideband ambiguity function, Sigmoid correlation, Sigmoid-MUSIC

## Abstract

Since second-order statistics-based methods rely heavily on Gaussianity assumption and fractional lower-order statistics-based methods depend on a priori knowledge of non-Gaussian noise, there remains a void in wideband bistatic multiple-input/multiple-output (MIMO) radar systems under impulsive noise. In this paper, a novel method based on Sigmoid transform was used to estimate target parameters, which do not need a priori knowledge of the noise in an impulsive noise environment. Firstly, a novel wideband ambiguity function, termed Sigmoid wideband ambiguity function (Sigmoid-WBAF), is proposed to estimate the Doppler stretch and time delay by searching the peak of the Sigmoid-WBAF. A novel Sigmoid correlation function is proposed. Furthermore, a new MUSIC algorithm based on the Sigmoid correlation function (Sigmoid-MUSIC) is proposed to estimate the direction-of-departure (DOD) and direction-of-arrival (DOA). Then, the boundness of the Sigmoid-WBAF to the symmetric alpha stable (SαS) noise, the feasibility analysis of the Sigmoid-WBAF, and complexity analysis of the Sigmoid-WBAF and Sigmoid-MUSIC are presented to evaluate the performance of the proposed method. In addition, the Cramér–Rao bound for parameter estimation was derived and computed in closed form, which shows that better performance was achieved. Simulation results and theoretical analyses are presented to verify the effectiveness of the proposed method.

## 1. Introduction

Recently, multiple-input/multiple-output (MIMO) systems attracted more and more attention [1,2,3]. There is rapidly growing literature concerning target parameter estimation in MIMO radar systems. Generally, target parameter estimation algorithms can be used in both narrowband and wideband MIMO radar systems. In narrowband MIMO radar systems, Yoe et al. proposed a computationally efficient method using properly designed projection filters to estimate the direction-of-departure (DOD) and direction-of-arrival (DOA) of targets [4]. Chen et al. proposed a method based on ESPRIT using the rotational factor produced by time-delay sampling to estimate the Doppler, DOD, and DOA [5]. Yao et.al. proposed a novel estimation method based on a non-uniform array configuration to estimate the DOD and DOA parameters, and analyzed their practical identifiability [6]. Jiang et al. proposed a joint estimation algorithm based on the canonical correlation decomposition (CCD) and exploits the shift-invariance properties in the Kronecker product structure of each column of the various steering matrices in unknown correlated noise [7]. Some improved algorithms based on MUSIC and ESPRIT were also proposed to estimate DOD and DOA parameters [8,9,10]. These methods were shown to yield good performance in terms of their parameters. In many applications, however, it is not appropriate to approximate the signal using a narrowband model.

In wideband radar systems, the echo from a wideband signal often contains a Doppler stretch (DS), not merely a Doppler shift, which results in parameter estimation difficulty [11,12]. To determine the range and relative velocity of a target, an accurate estimation of these parameters is crucial. Ma et al. proposed two novel methods for incoherent broadband chirp DOA estimation (BCD-I) and coherent broadband chirp DOA estimation (BCD-C) [13]. You et al. and Xu et al. presented DOA estimation methods of wideband signal based on spectral correlation signal subspace fitting (SC-SSF) [14,15]. Chen et al. proposed a method of DOA estimation for wideband coherent acoustic sources based on coherent signal subspace [16], and Yoon [17] presented a DOA estimation method based on the orthogonality of projected subspaces to estimate DOA. However, these methods did not estimate the Doppler shift and time delay, which are also very crucial for the determination of the range and velocity of the target in a wideband bistatic MIMO radar. At present, we seldom find the study of a joint estimation for the Doppler shift, time delay, DOD, and DOA in a wideband bistatic MIMO radar [18], which needs to be studied deeply because of its usage for target tracking and target localization.

In general, broadband chirp signals are frequently used in sonar and radar systems. The broadband chirp or linear frequency-modulated (LFM) signals are known to be insensitive to the Doppler of echoes and have better properties of low probability of interception. Therefore, in this paper, we utilized an LFM signal as a transmitted signal to study the wideband signal model parameter estimation in bistatic MIMO radars.

Until now, in most parameter estimation methods for array signal processing, additive noise is assumed to be Gaussian. Studies and experimental measurements showed that broad classes of noise such as underwater acoustic noise, atmospheric noise, multiuser interference, and radar clutters in real-world applications are non-Gaussian, primarily owing to impulsive phenomena [19,20,21,22,23,24,25,26]. Taking these scenarios into account, it is inappropriate to model the noise as Gaussian noise. Researchers studied this impulsive nature and showed that symmetric alpha stable (SαS) processes are better models for impulsive noise than Gaussian processes. Conventional algorithms based on second-order statistics degenerate severely in an impulsive noise environment [12,21].

To reduce the alpha stable distribution noise interference, many parameter estimation algorithms based on the fractional lower-order statistics (FLOS) were proposed [18,19,20,21,22,23,24,25,26,27]. However, these algorithms have some limitations: (1) the noise characteristic exponent must be estimated to ensure 1≤p<α or 0<p<α/2, where p is the fractional lower order of moments and α is the characteristic exponent of the impulsive noise; (2) these methods cannot accurately estimate the parameters if there is no a priori knowledge of the characteristic exponent. Furthermore, the performance of these algorithms can degrade seriously and even become invalid when the fractional lower-order moment value is not appropriate. To handle this problem, a novel wideband ambiguity function based on Sigmoid transform, referred to as Sigmoid wideband ambiguity function (Sigmoid-WBAF), is proposed to estimate the Doppler stretch (DS) and time delay (TD) in this paper. Then, a novel correlation function based on the Sigmoid transform, referred to as Sigmoid correlation, is defined. Furthermore, a novel MUSIC method based on the Sigmoid correlation, referred as Sigmoid-MUSIC, is proposed to estimate the DOD and DOA.

This paper is organized as follows: Section 2 presents a signal model of a wideband bistatic MIMO radar system. Section 3 describes a novel Sigmoid wideband ambiguity function and a novel Sigmoid correlation. In Section 4, the Sigmoid-WBAF and Sigmoid-MUSIC methods are used to estimate the target parameters. In Section 5, the boundness of the Sigmoid-WBAF to the symmetric alpha stable (SαS) noise, and the feasibility analysis of the Sigmoid-WBAF and the Cramér–Rao bound for parameter estimation are presented to evaluate the performance of the proposed method. In Section 6, the performance of the parameter estimation algorithm is studied through extensive numerical simulations. Finally, conclusions are drawn in Section 6.

## 2. Signal Model and Noise Model

### 2.1. Signal Model and Bandpass Matched Filter

#### 2.1.1. Signal Model

**Figure 1:**
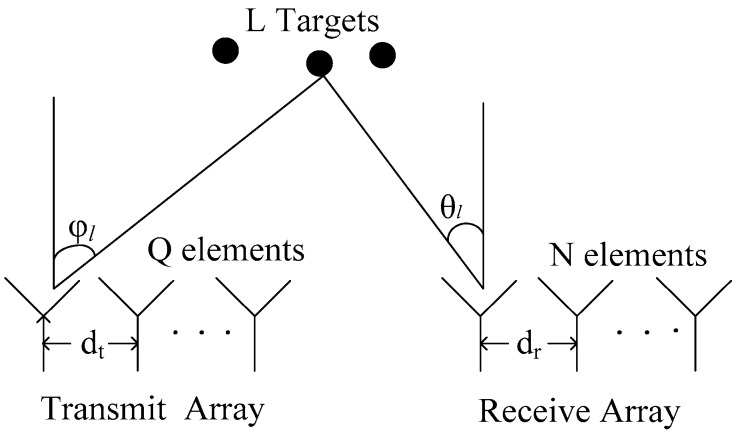
Bistatic multiple-input/multiple-output (MIMO) radar system.

Consider a bistatic MIMO radar system with a Q-transmitter and an N-receiver, both of which are half-wavelength spaced uniform linear arrays. Assume that there are L uncorrelated targets in the far field, where the targets are located, and the signal wavefront is considered as a plane wave. The described bistatic MIMO radar system is illustrated as a two-dimensional (2D) configuration in Figure 1.

Each antenna transmits the linear frequency modulation signal $x_{q}(t)${q=1,…,Q}. These transmitted signals can be reflected by *L* targets at positions $\left(\varphi_{l},\theta_{l}\right)$ {l=1,…,L}, where $\varphi_{l}$ denotes the DOD and $\theta_{l}$ denotes the DOA. In wideband sonar and radar systems, the echo from a wideband signal often contains a Doppler stretch (DS), in addition to a Doppler shift, due to the moving of the target. Thus, the received signal $y_{n}\left(t\right)$ at the n-th receive antenna can be described as follows [18]:

(1) $$y_{n}\left(t\right)=\begin{array}{c} \sum_{l=1}^{L}\sum_{q=1}^{Q}\beta_{l}x_{q}\left(\frac{t-\tau_{l}}{\sigma_{l}}\right)B_{q}\left(\varphi_{l}\right)A_{n}\left(\theta_{l}\right)+w_{n}\left(t\right) \end{array},$$

where $x_{q}(t)$ is the transmitted signal by the q-th transmit antenna, expressed as

(2) $$x_{q}(t)=A_{q}\exp\left[j2\pi \left(f_{q0}t+\mu_{q0}t^{2}/2\right)\right],$$

where $A_{q}$ is the amplitude of the transmitted signal, $f_{q0}$ and $\mu_{q0}$ are the initial frequency and the chirp rate, respectively, $\beta_{l}$ is the radar cross-section (RCS), $\sigma_{l}$ is the Doppler stretch, $\tau_{l}$ is the time delay, $B_{q}\left(\varphi_{l}\right)=\exp\left(j\pi \left(q-1\right)\sin\varphi_{l}\right)$ is the *q*-th element of the transmitter steering vector, $A_{n}\left(\theta_{l}\right)=\exp\left(j\pi \left(n-1\right)\sin\theta_{l}\right)$ is the n-th element of the receiver steering vector, and $w_{n}\left(t\right)$ is a sequence of independent and identically distributed (i.i.d.) isotropic complex SαS random variables.

#### 2.1.2. Bandpass Matched Filter

The fractional Fourier transform (FRFT) is a generalization of the FT, and can be interpreted as a rotation of the signal to any angles in the time–frequency plane [28]. The continuous FRFT of a signal f(t) with angle ρ is defined as

(3) $$F\left(\rho ,m\right)=F^{\rho }\left[f\left(t\right)\right]\left(m\right)=\int_{-\infty }^{+\infty }f\left(t\right)K_{\rho }\left(t,m\right)dt,$$

where $F^{\rho }$ denotes the FRFT operator and $K_{\rho }(t,m)$ is the kernel function of the fractional Fourier transform. $K_{\rho }(t,m)$ can be expressed as

(4) $$K_{\rho }(t,m)=\left\{ \begin{array}{l} \sqrt{\left(1-j\cot\rho \right)}\exp\left(j\pi (t^{2}\cot\rho -2mt\csc\rho +m^{2}\cot\rho )\right),\rho \neq n\pi \\ \delta (t-m),\;\rho =2n\pi \\ \delta (t+m),\;\rho =(2n+1)\pi \end{array}\right.,$$

where ρ and m are the rotation angle and the frequency in the FRFT domain, respectively.

According to Equations (2) and (3), the FRFT of transmitted signal $x_{q}(t)$ with an angle ρ is defined as

(5) $$\begin{array}{l} X_{q}\left(\rho ,m\right)=A\sqrt{\left(1-j\cot\rho \right)}\exp\left(j\pi m^{2}\cot\rho \right)\\ \;\cdot \int_{0}^{T}\exp\left(j2\pi t\left(f_{q0}-m\csc\rho \right)\right)\exp\left(j\pi t^{2}\left(\cot\rho +\mu_{q0}\right)\right)dt. \end{array}.$$

$X_{q}\left(\rho ,m\right)$ produces the peak value when $\mu_{q0}=-\cot\rho_{q0}$ and $f_{q0}=m_{q0}\csc\rho_{q0}$.

The FRFT $X_{q}\left(\rho_{q0},m\right)$ of signal $x_{q}(t)$ with optimal angle $\rho_{q0}$ has an obvious peak value, and the energy of $X_{q}\left(\rho_{q0},m\right)$ concentrates in a narrow band with the central frequency of $m_{q0}$.

A q-th bandpass matched filter with suitable bandwidth and central frequency $m_{q0}$ is designed. Let $R_{qn}\left(\alpha ,m\right)$ denote the output of the q-th matched filter at the n-th receive antenna. $y_{qn}\left(t\right)$ represents the output of the matched filter in the FRFT domain, which corresponds to the q-th transmit antenna signal in the *n*-th receive antenna. Through the inverse fractional Fourier transformation of $R_{qn}\left(\alpha ,m\right)$, $y_{qn}\left(t\right)$ can be expressed as

(6) $$y_{qn}\left(t\right)=\sum_{l=1}^{L}\beta_{l}x_{q}\left(\frac{t-\tau_{l}}{\sigma_{l}}\right)A_{n}\left(\theta_{l}\right)B_{q}\left(\varphi_{l}\right)+w_{qn}\left(t\right),$$

where $w_{qn}\left(t\right)$ denotes the SαS noise in the received signal $y_{qn}\left(t\right)$.

### 2.2. SαS Distribution Noise Model

Symmetric α stable (SαS) processes are the subject of increased attention as a suitable framework for efficient signal processing in impulsive environments [19,20,29,30]. A “zero-centered” symmetric α stable distribution is commonly described through its characteristic function as

(7) $$\psi \left(\omega \right)=\exp\left(-\gamma {\left|\omega \right|}^{\alpha }\right),$$

where parameter α is usually called the characteristic exponent. It can be proven that, in order to define a characteristic function for Equation (7), the values of α must be restricted to the interval (0,2]. When α<2, the distribution is algebraic-tailed with tail constant α, implying infinite variance. When α=2, the *SαS* distribution reduces to the Gaussian distribution implying lighter-than-algebraic tails. The parameter γ, usually called the dispersion, is a positive constant related to the scale of the distribution. For a fixed α, larger values of γ correspond to larger strengths of the process. It is easy to see that $\gamma^{1/\alpha }$ is, in fact, a scale parameter of the distribution.

Let X be a logarithmic-order random variable. We define the geometric power of X as follows [23]:

(8) $$S_{0}=S_{0}\left(X\right)=\exp\left(E\left[\log\left|X\right|\right]\right).$$

The geometric power of a symmetric α stable random variable as defined in Equation (7) is given by

(9) $$S_{0}={\left(C_{g}\gamma \right)}^{1/\alpha }/C_{g},$$

where $C_{g}\approx 1.78$ is the exponential of the Euler constant.

Since the α stable distribution with α<2 determines an infinite variance, we describe the signal-to-noise condition of SαS using the generalized signal-noise-ratio (GSNR) [19,29,30], which is defined as

(10) $$\mathrm{GSNR}=10\mathrm{lg}\left(\sigma_{x}^{2}/\gamma \right),$$

where $\sigma_{x}^{2}$ is the variance of the underlying signal.

## 3. Sigmoid Wideband Ambiguity Function and Sigmoid Correlation

### 3.1. Wideband Ambiguity Function

For a joint estimation of TD and DS, Swick [31,32] suggested the application of a wideband ambiguity function (WBAF), defined by

(11) $$W_{s_{r}s}\left(\tau ,\sigma \right)=\frac{1}{\sqrt{\sigma }}\int_{-\infty }^{\infty }s_{r}\left(t\right)\;s^{*}\left(\frac{t-\tau }{\sigma }\right)dt,$$

where $s_{r}\left(t\right)=s\left(\frac{t-\tau_{0}}{\sigma_{0}}\right)$, and $\tau_{0}$ and $\sigma_{0}$ are the time delay and Doppler stretch, respectively.

From the Schwarz inequality, we can see that

(12) $$\begin{array}{ll} {\left|W_{s_{r}s}\left(\tau ,\sigma \right)\right|}^{2} & =\frac{1}{\sigma }{\left|\int_{-\infty }^{\infty }s_{r}\left(t\right)\;s^{*}\left(\frac{t-\tau }{\sigma }\right)dt\right|}^{2}\\ & =\frac{1}{\sigma }{\left|\int_{-\infty }^{\infty }s_{r}\left(\frac{t-\tau_{0}}{\sigma_{0}}\right)\;s^{*}\left(\frac{t-\tau }{\sigma }\right)dt\right|}^{2}\\ & \leq \left[{\int_{-\infty }^{\infty }\left|s_{r}\left(\frac{t-\tau_{0}}{\sigma_{0}}\right)\right|}^{2}dt\right]\left[\frac{1}{\sigma }{\int_{-\infty }^{\infty }\left|s\left(\frac{t-\tau }{\sigma }\right)\right|}^{2}dt\right] \end{array}.$$

Equation (12) satisfies equality if and only if $s\left(\frac{t-\tau }{\sigma }\right)\propto s_{r}\left(\frac{t-\tau_{0}}{\sigma_{0}}\right)$ is equivalent to $\tau =\tau_{0}$ and $\sigma =\sigma_{0}$ when σ>0 σ>0 and $\sigma_{0}>0$. Since ${\left|W_{s_{r}s}\left(\tau ,\sigma \right)\right|}^{2}$ attains its maximum only at $\tau =\tau_{0}$ and $\sigma =\sigma_{0}$, the estimation of TD and DS becomes a problem in locating the maximum point of ${\left|W_{s_{r}s}\left(\tau ,\sigma \right)\right|}^{2}$, and the corresponding coordinate is the true TD and DS point $\left(\tau_{0},\sigma_{0}\right)$ and can be depicted as follows [12]:

(13) $$\left(\tau =\tau_{0},\sigma =\sigma_{0}\right)=\arg\max\left|W_{s_{r}s}\left(\tau ,\sigma \right)\right|.$$

According to Equation (11), we obtain the wideband ambiguity function $W_{x_{r,qnl}x_{q}}\left(\tau ,\sigma \right)$ of $x_{q}\left(t\right)$ and $x_{r,qnl}\left(t\right)$ as

(14) $$W_{x_{r,qnl}x_{q}}\left(\tau ,\sigma \right)=\frac{1}{\sqrt{\sigma }}\int_{-\infty }^{\infty }x_{r,qnl}\left(t\right)x_{q}^{*}\left(\frac{t-\tau }{\sigma }\right)dt,$$

where $x_{r,qnl}\left(t\right)=\beta_{l}x_{q}\left[\left(t-\tau_{l}\right)/\sigma_{l}\right]B_{q}\left(\varphi_{l}\right)A_{n}\left(\theta_{l}\right)$, l denotes the l-th target, and l=1,…,L.

From the Schwarz inequality, we can also see that

(15) $$\begin{array}{ll} {\left|W_{x_{r,qnl}x_{q}}\left(\tau ,\sigma \right)\right|}^{2} & =\frac{1}{\sigma }{\left|\int_{-\infty }^{\infty }x_{r,qnl}\left(t\right)x_{q}^{*}\left(\frac{t-\tau }{\sigma }\right)dt\right|}^{2}\\ & \leq {\left|\beta_{l}\right|}^{2}\left[{\int_{-\infty }^{\infty }\left|x_{q}\left(\frac{t-\tau_{l}}{\sigma_{l}}\right)\right|}^{2}dt\right]\left[\frac{1}{\sigma }{\int_{-\infty }^{\infty }\left|x_{q}\left(\frac{t-\tau }{\sigma }\right)\right|}^{2}dt\right] \end{array}.$$

Equation (15) holds with equality if and only if $x_{q}\left(\frac{t-\tau }{\sigma }\right)\propto x_{q}\left(\frac{t-\tau_{l}}{\sigma_{l}}\right)$ is equivalent to $\tau =\tau_{l}$ and $\sigma =\sigma_{l}$ when σ>0 and $\sigma_{l}>0$. Since ${\left|W_{x_{r,qnl}x_{q}}\left(\tau ,\sigma \right)\right|}^{2}$ attains its maximum only at $\tau =\tau_{l}$ and $\sigma =\sigma_{l}$, the estimation of TD and DS becomes a problem in locating the maximum point of ${\left|W_{x_{r,qnl}x_{q}}\left(\tau ,\sigma \right)\right|}^{2}$, and the corresponding coordinate is the true TD and DS point $\left(\tau_{l},\sigma_{l}\right)$.

Then, it follows directly from Equation (16) that the TD and DS are determined by

(16) $$\begin{array}{l} \left(\tau_{l},\sigma_{l}\right)=\arg\underset{\tau ,\sigma }{\max}\left[\left|W_{x_{r,qnl}x_{q}}\left(\tau ,\sigma \right)\right|\right]\\ W_{x_{r,qnl}x_{q}}\left(\tau_{l},\sigma_{l}\right)=\max\left[\left|W_{x_{r,qnl}x_{q}}\left(\tau ,\sigma \right)\right|\right] \end{array}\}.$$

When the alpha stable distribution noise is added, this peak location algorithm may fail. The reason is that the alpha stable distribution does not have a finite α-order moment and other higher-than-α-order moments, and the wideband ambiguity function is based on a second-order moment. Accordingly, the WBAF algorithm becomes unbounded when the received noise contains a α-stable distribution noise. Therefore, we present a nonlinear transform, the Sigmoid transform, to suppress the α stable distribution noise interference.

### 3.2. Sigmoid Transform

Sigmoid is a commonly used nonlinear transform [33,34,35]. Its definition is shown in Equation (17).

(17) $$\mathrm{Sigmoid}\left[x\left(t\right)\right]=\frac{2}{1+\exp\left[-x\left(t\right)\right]}-1.$$

Two properties can be obtained from the analysis of the Sigmoid function transform.

**Property** **1.** *If* x(t) *is an* SαS *process with* β=0 *and* a=0 *, then* Sigmoid[x(t)] *is a symmetric distribution with zero mean in its probability density function, and has the finite second-order moment with zero mean (referred to as a second-order moment process).*

**Property** **2.** *Set*X(t)=Sigmoid[x(t)]*; then,*X(t)*has the same frequency shift as*x(t).

Since Properties 1 and 2 were proven in References [35] and [29,30], respectively, the relevant proof is skipped herein.

### 3.3. Sigmoid Wideband Ambiguity Function

A novel ambiguity function based on the Sigmoid transform, the Sigmoid wideband ambiguity function (referred to as Sigmoid-WBAF), $W_{s_{r}s}^{\mathrm{Sigmoid}}\left(\tau ,\sigma \right)$, is defined as

(18) $$W_{s_{r}s}^{\mathrm{Sigmoid}}\left(\tau ,\sigma \right)=\frac{1}{\sqrt{\sigma }}\int_{-\infty }^{+\infty }\mathrm{Sigmoid}\left[s_{r}\left(t\right)\right]\;{\mathrm{Sigmoid}}^{*}\left[s\left(\frac{t-\tau }{\sigma }\right)\right]dt.$$

This is the form of Sigmoid-WBAF used throughout this paper. Note that $W_{s_{r}s}^{\mathrm{Sigmoid}}\left(\tau ,\sigma \right)$ is a 2D representation of the Sigmoid correlation between $s_{r}\left(t\right)$ and $s\left(\frac{t-\tau }{\sigma }\right)$ for various values of τ and σ.

We note that, in Equation (18), the limits of the integral are from −∞ to ∞. However, in practice, we only use signals which are essentially time-limited to [−T/2,T/2], meaning that the signal amplitude is negligible outside the essential duration. Thus, the practical implication of employing Equation (13) is that it will be applied only during the essential duration of [−T/2,T/2]. Also, in practice and for a time finite signal, $W_{s_{r}s}^{\mathrm{Sigmoid}}\left(\tau ,\sigma \right)$ can be estimated by Equation (19).

(19) $${\hat{W}}_{s_{r}s}^{\mathrm{Sigmoid}}\left(\tau ,\sigma \right)=\frac{1}{\sqrt{\sigma }}\int_{-T/2}^{T/2}\mathrm{Sigmoid}\left[s_{r}\left(t\right)\right]\;{\mathrm{Sigmoid}}^{*}\left[s\left(\frac{t-\tau }{\sigma }\right)\right]dt.$$

Similarly, the joint estimates of TD and DS can be obtained by

(20) $$\begin{array}{r} \left(\tau_{0},\sigma_{0}\right)=\arg\underset{\tau ,\sigma }{\max}\left[\left|{\hat{W}}_{s_{r}s}^{\mathrm{Sigmoid}}\left(\tau ,\sigma \right)\right|\right]\\ {\hat{W}}_{s_{r}s}^{\mathrm{Sigmoid}}\left(\tau_{0},\sigma_{0}\right)=\max\left[\left|{\hat{W}}_{s_{r}s}^{\mathrm{Sigmoid}}\left(\tau ,\sigma \right)\right|\right] \end{array}\}.$$

### 3.4. Sigmoid Correlation

For an alpha stable distribution noise environment, the conditional MUSIC algorithm performance degrades seriously and even becomes invalid because the conditional MUSIC algorithm is based on a second-order moment. To suppress the alpha stable distribution noise interference, a novel correlation function is proposed in this section.

A novel correlation based on the Sigmoid transform $R_{x}^{\mathrm{Sigmoid}}\left(\tau \right)$, referred to as the Sigmoid correlation (SC), is defined in Equation (21).

(21) $$\begin{array}{ll} R_{x}^{\mathrm{Sigmoid}}\left(\tau \right) & =\int_{-T/2}^{T/2}\mathrm{Sigmoid}\left[x\left(t\right)\right]\;{\mathrm{Sigmoid}}^{*}\left[x\left(t-\tau \right)\right]\;dt\\ & ={\langle \mathrm{Sigmoid}\left[x\left(t\right)\right]\;{\mathrm{Sigmoid}}^{*}\left[x\left(t-\tau \right)\right]\rangle }_{t} \end{array},$$

where ${\langle \cdot \rangle }_{t}$ represents a time average.

According to the properties of the Sigmoid transform, we can deduce that the properties of correlation do not change if the Sigmoid transform is applied on both signals in advance. Therefore, the Sigmoid correlation function not only has the properties of a conventional autocorrelation function, but also has a suppression ability to impulsive noise. Therefore, the Sigmoid correlation function can be used to estimate DOD and DOA in an impulsive noise environment.

### 3.5. Sigmoid-MUSIC Algorithm

Let

(22) y(t)=As(t)+n(t),

where $y\left(t\right)={\left[y_{1}\left(t\right)\;y_{2}\left(t\right)\;\cdots y_{M}\left(t\right)\right]}^{T}$ is the M×1 vector of signals received by the array sensors, $s\left(t\right)={\left[s_{1}\left(t\right)\;s_{2}\left(t\right)\cdots s_{P}\left(t\right)\right]}^{T}$ is the P×1 vector of the signals, $n\left(t\right)={\left[n_{1}\left(t\right)\;n_{2}\left(t\right)\cdots n_{P}\left(t\right)\right]}^{T}$ is the M×1 vector of impulsive noise, and $A=\left[a\left(\theta_{1}\right)\;\cdots a\left(\theta_{i}\right)\cdots a\left(\theta_{P}\right)\right]$ is the matrix of array steering vectors, in which $a\left(\theta_{i}\right)=\left[1\cdots e^{j2\pi \left(m-1\right)d\sin\theta_{i}/\lambda }\cdots e^{j2\pi \left(M-1\right)d\sin\theta_{i}/\lambda }\right]$.

The Sigmoid-MUSIC algorithm can be implemented using the following procedure:

Step 1. Compute Sigmoid correlation matrices $R_{y}^{\mathrm{Sigmoid}}\left(\tau \right)$ of the matrix y(t), according to Equation (21).
Step 2. Execute singular value decomposition (SVD) on $R_{y}^{\mathrm{Sigmoid}}\left(\tau \right)$, where the column vector $U_{N}$ describes the eigenvectors spanning the noise subspace.
Step 3. Compute the corresponding Sigmoid-MUSIC spectrum $P_{\mathrm{Sigmoid}-\mathrm{MUSIC}}\left(\theta \right)$ as
  (23) $$P_{\mathrm{Sigmoid}-\mathrm{MUSIC}}\left(\theta \right)=\frac{1}{A^{H}U_{N}U_{N}^{H}A}.$$
Step 4. The estimator of θ can be obtained by searching for peaks of the Sigmoid-MUSIC spectrum $P_{\mathrm{Sigmoid}-\mathrm{MUSIC}}\left(\theta \right)$.

## 4. Joint Estimation Parameter Based on Sigmoid-WBAF and Sigmoid-MUSIC

In this section, a study of parameter estimation is made by considering signal $y_{qnl}\left(t\right)$ as an example. Signal $y_{qnl}\left(t\right)$ denotes the received signals $y_{qn}\left(t\right)$ corresponding to the l-th target. $y_{qnl}\left(t\right)$ can be expressed as

(24) $$y_{qnl}\left(t\right)=\beta_{l}x\left(\frac{t-\tau_{l}}{\sigma_{l}}\right)A_{n}\left(\theta_{l}\right)B_{q}\left(\varphi_{l}\right)+w_{qnl}\left(t\right),$$

where $w_{qnl}\left(t\right)$ denotes the SαS noise in the received signal $y_{qnl}\left(t\right)$.

### 4.1. Estimation of TD and DS Based on Sigmoid-WBAF

According to Equations (2), (19), and (24), we can obtain the Sigmoid-WBAF of $y_{qnl}\left(t\right)$ and $x_{q}\left(t\right)$ as follows:

(25) $$\begin{array}{ll} {\hat{W}}_{y_{qnl}x_{q}}^{\mathrm{Sigmoid}}\left(\tau ,\sigma \right) & =\frac{1}{\sqrt{\sigma }}\int_{-T/2}^{T/2}\mathrm{Sigmoid}\left[y_{qnl}\left(t\right)\right]\;{\mathrm{Sigmoid}}^{*}\left[x_{q}\left(\frac{t-\tau }{\sigma }\right)\right]dt\\ & ={\hat{W}}_{x_{r,qnl}x_{q}}^{\mathrm{Sigmoid}}\left(\tau ,\sigma \right)+{\hat{W}}_{w_{qnl}x_{q}}^{\mathrm{Sigmoid}}\left(\tau ,\sigma \right) \end{array},$$

where ${\hat{W}}_{w_{qnl}x_{q}}^{\mathrm{Sigmoid}}\left(\tau ,\sigma \right)$ is the Sigmoid wideband ambiguity function of noise $w_{qnl}\left(t\right)$ and transmitted signal $x_{q}\left(t\right)$, and it is treated as a random interference.

Similarly, the estimators of TD and DS can be obtained from

(26) $$\begin{array}{r} \left(\tau ={\hat{\tau }}_{l},\sigma ={\hat{\sigma }}_{l}\right)=\arg\underset{\tau ,\sigma }{\max}\left[\left|{\hat{W}}_{y_{qnl}x_{q}}^{\mathrm{Sigmoid}}\left(\tau ,\sigma \right)\right|\right]\\ {\hat{W}}_{y_{qnl}x_{q}}^{\mathrm{Sigmoid}}\left({\hat{\tau }}_{l},{\hat{\sigma }}_{l}\right)=\max\left[\left|{\hat{W}}_{y_{qnl}x_{q}}^{\mathrm{Sigmoid}}\left(\tau ,\sigma \right)\right|\right] \end{array}\}.$$

Accordingly, the estimation of the Doppler stretch and time delay in a wideband bistatic MIMO radar was achieved via the proposed Sigmoid wideband ambiguity function. The steps involved in this process were as follows:

Step 1. Present the extracted signal $y_{qn}\left(t\right)$.
Step 2. Compute the Sigmoid-WBAF function ${\hat{W}}_{y_{qn}x_{q}}^{\mathrm{Sigmoid}}\left(\tau ,\sigma \right)$ from Equation (25).
Step 3. Search for the peaks of ${\hat{W}}_{y_{qn}x_{q}}^{\mathrm{Sigmoid}}\left(\tau ,\sigma \right)$ and obtain the locations of these peaks $\left({\hat{\tau }}_{l},{\hat{\sigma }}_{l}\right)$, for l=1,…,L.
Step 4. Estimate the DS and TD according to Equation (26).

### 4.2. Estimation of DOD and DOA Based on Sigmoid-MUSIC

In this section, DOD and DOA are estimated by employing the proposed Sigmoid-MUSIC.

The vector form of the array output can be shown as

(27) Y(t)=A⊗BS+N(t),

where $A=\left[a_{1},\ldots ,a_{L}\right]$ and $B=\left[b_{1},\ldots ,b_{L}\right]$ are the receive and transmit steering matrices, where the receiver and transmitter steering vectors are given by $a_{l}={\left[1,e^{j\pi \sin\theta_{l}},\ldots ,e^{j\pi \left(N-1\right)\sin\theta_{l}}\right]}^{T}$ and $b_{l}={\left[1,e^{j\pi \sin\varphi_{l}},\ldots ,e^{j\pi \left(Q-1\right)\sin\varphi_{l}}\right]}^{T}$. $S=\left[s_{1},\ldots ,s_{L}\right]$, where $s_{l}={\left[\beta_{l}x_{1}\left(\frac{t-\tau_{l}}{\sigma_{l}}\right),\ldots ,\beta_{l}x_{Q}\left(\frac{t-\tau_{l}}{\sigma_{l}}\right)\right]}^{T}$ is a known vector since estimated values of $\tau_{l}$ and $\sigma_{l}$ are used, ⊗ denotes the Kronecker Matrix product, and N(t) is a sequence of i.i.d isotropic complex SαS random variables.

According to Equation (27), two receive subarrays $Y_{1}$ and $Y_{2}$ can be constructed as follows:

(28) $$Y_{1}=AS+N_{1}\;q=1;$$

(29) $$Y_{2}=BS+N_{2}\;n=1.$$

According to Equation (21), we can obtain the Sigmoid correlation function $R_{Y_{1}}^{\mathrm{Sigmoid}}\left(\tau \right)$ of the received signal vector $Y_{1}\left(t\right)$,

(30) $$\begin{array}{ll} R_{Y_{1}}^{\mathrm{Sigmoid}}\left(\tau \right) & ={\langle \mathrm{Sigmoid}\left[Y_{1}\left(t\right)\right]\;{\mathrm{Sigmoid}}^{*}\left[Y_{1}\left(t-\tau \right)\right]\rangle }_{t}\\ & =AR_{S}^{\mathrm{Sigmoid}}\left(\tau \right)A^{H}+AR_{SN^{*}}^{\mathrm{Sigmoid}}\left(\tau \right)+R_{S^{*}N}^{\mathrm{Sigmoid}}\left(\tau \right)A^{H}+R_{NN^{*}}^{\mathrm{Sigmoid}}\left(\tau \right) \end{array},$$

where $R_{S}^{\mathrm{Sigmoid}}\left(\tau \right)={\langle \mathrm{Sigmoid}\left[s\left(t\right)\right]{\mathrm{Sigmoid}}^{*}\left[s\left(t-\tau \right)\right]\rangle }_{t}$ denotes the Sigmoid autocorrelation of the matrix S. Note that the correlation matrix used in this algorithm is replaced by the Sigmoid correlation matrix.

Signal s(t) is independent of noise n(t); thus, Equation (30) can be rewritten as

(31) $$R_{Y_{1}}^{\mathrm{Sigmoid}}\left(\tau \right)=AR_{S}^{\mathrm{Sigmoid}}\left(\tau \right)A^{H}.$$

Equation (31) can then be rewritten as

(32) $$R_{Y_{1}}^{\mathrm{Sigmoid}}\left(\tau \right)=\left[\begin{array}{cc} U_{S} & U_{N} \end{array}\right]\left[\begin{array}{cc} \sum_{S} & 0\\ 0 & \sum_{N} \end{array}\right]{\left[\begin{array}{cc} V_{S} & V_{N} \end{array}\right]}^{H}=U_{S}\sum_{S}V_{S}^{H},$$

where $\left[\begin{array}{cc} U_{S} & U_{N} \end{array}\right]$ and $\left[\begin{array}{cc} V_{S} & V_{N} \end{array}\right]$ are unitary, and the diagonal elements of the diagonal matrix $\sum_{S}$ are positive. The column vectors of $U_{S}$ and $U_{N}$ are the eigenvectors spanning the signal subspace and noise subspace of $R_{Y_{1}}^{\mathrm{Sigmoid}}\left(\tau \right)$, respectively, with the associated eigenvalues as the diagonals of $\sum_{S}$ and $\sum_{N}$. Since signal S is independent of the noise N,

(33) $$R_{S}^{\mathrm{Sigmoid}}\left(\tau \right)U_{N}=0,$$

and the signal and noise subspaces are orthogonal, i.e.,

(34) $$A^{H}U_{N}=0.$$

Therefore, the spatial spectrum of Sigmoid-MUSIC can be obtained based on the classical MUSIC algorithm, which can be expressed as

(35) $$P_{Y_{1}}\left(\theta \right)=\frac{1}{A^{H}U_{N}U_{N}^{H}A}.$$

Searching for the spectral peak of $P_{Y_{1}}\left(\theta \right)$, we can get the DOA estimator $\theta_{l}$.

Similarly, we can obtain the Sigmoid correlation function $R_{Y_{2}}^{\mathrm{Sigmoid}}\left(\tau \right)$ of the received signal vector $Y_{2}\left(t\right)$ according to Equations (29)–(32) as

(36) $$R_{Y_{2}}^{\mathrm{Sigmoid}}\left(\tau \right)=\left[\begin{array}{cc} U_{S_{2}} & U_{N_{2}} \end{array}\right]\left[\begin{array}{cc} \sum_{S_{2}} & 0\\ 0 & \sum_{N_{2}} \end{array}\right]{\left[\begin{array}{cc} V_{S_{2}} & V_{N_{2}} \end{array}\right]}^{H}=U_{S_{2}}\sum_{S_{2}}V_{S_{2}}^{H},$$

where $\left[\begin{array}{cc} U_{S_{2}} & U_{N_{2}} \end{array}\right]$ and $\left[\begin{array}{cc} V_{S_{2}} & V_{N_{2}} \end{array}\right]$ are unitary, and the diagonal elements of the diagonal matrix $\sum_{S_{2}}$ are positive. The column vectors of $U_{S_{2}}$ and $U_{N_{2}}$ are the eigenvectors spanning the signal subspace and noise subspace of $R_{Y_{2}}^{\mathrm{Sigmoid}}\left(\tau \right)$, respectively, with the associated eigenvalues as the diagonals of $\sum_{S_{2}}$ and $\sum_{N_{2}}$.

Therefore, the Sigmoid-MUSIC algorithm was employed on matrix $Y_{2}$; we can also obtain the spatial spectrum of Sigmoid-MUSIC for the subarray $Y_{2}$ as

(37) $$P_{Y_{2}}\left(\varphi \right)=\frac{1}{B^{H}U_{N_{2}}U_{N_{2}}^{H}B}.$$

Searching for the spectral peak of $P_{Y_{2}}\left(\varphi \right)$, we can get the DOD estimator $\varphi_{l}$.

Accordingly, the estimation of DOD and DOA in a wideband bistatic MIMO radar was achieved via the proposed Sigmoid-MUSIC algorithm. The steps involved in this process were as follows:

Step 1. Construct two matrices $Y_{1}$ and $Y_{2}$.
Step 2. Substitute the time average with the statistic average, two Sigmoid correlation matrices $R_{Y_{1}}^{\mathrm{Sigmoid}}\left(\tau \right)$ and $R_{Y_{2}}^{\mathrm{Sigmoid}}\left(\tau \right)$ are then constructed according to Equation (21).
Step 3. Apply the singular value decomposition (SVD) to $R_{Y_{1}}^{\mathrm{Sigmoid}}\left(\tau \right)$ and $R_{Y_{2}}^{\mathrm{Sigmoid}}\left(\tau \right)$, where the column vectors $U_{N}$ and $U_{N_{2}}$ are formed from the eigenvectors spanning the noise subspace.
Step 4. Compute the corresponding Sigmoid-MUSIC spectra $P_{Y_{1}}\left(\theta \right)$ and $P_{Y_{2}}\left(\varphi \right)$ from Equations (36) and (37).
Step 5. The DOA and DOD estimates can be obtained by identifying the peaks of the spatial spectra $P_{Y_{1}}\left(\theta \right)$ and $P_{Y_{2}}\left(\varphi \right)$.

## 5. Analysis of Sigmoid-WBAF and Sigmoid-MUSIC

### 5.1. Boundness of Sigmoid-WBAF

According to the properties of the Sigmoid transform, the *SαS* process with a=0 can be transformed to a second-order moment process by the Sigmoid transform. Therefore, ${\hat{W}}_{y_{qnl}x_{q}}^{\mathrm{Sigmoid}}\left(\tau ,\sigma \right)$ is bounded for the SαS process because it is only involved with Sigmoid[x(t)] under the SαS noise. Furthermore, the transformation does not change the estimation results of the time delay and Doppler frequency shift. Therefore, the Sigmoid-WBAF method can be used to estimate the parameters of wideband echoes y(t) under SαS stable distribution noise.

### 5.2. Feasibility Analysis of Sigmoid-WBAF

**Figure 2:**
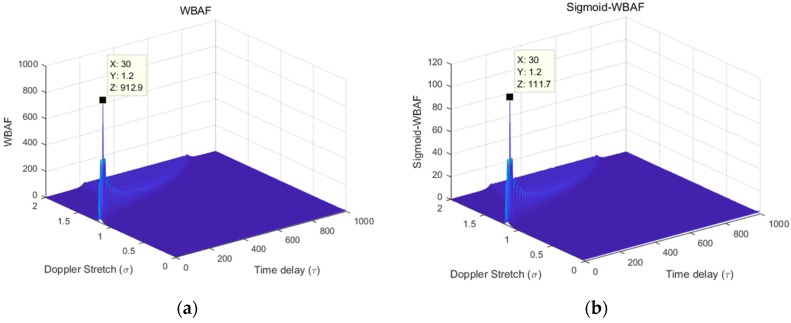
The spectra of (**a**) wideband ambiguity function (WBAF) and (**b**) Sigmoid-WBAF.

Simulation results and theoretical analyses illustrated that the Sigmoid transform does not change the modulation characteristics of the signal [29]. Therefore, the characteristics of WBAF do not change if the Sigmoid transform is applied to the signal in advance. This simulation result is illustrated in Figure 2 to verify this property. Figure 2 shows the spectra of WBAF and Sigmoid-WBAF.

From Figure 2, the WBAF of the LFM signal and Sigmoid-WBAF of the LFM signal have the same peak location in the WBAF domain. Therefore, the parameters of the Doppler stretch and time delay can be estimated by searching for the peak of Sigmoid-WBAF.

### 5.3. The Cramer–Rao Bound

In this section, we derive a novel explicit expression for the exact Cramer–Rao Bound (CRB) on the accuracy of estimating the signal model parameters [29].

The received signal can be expressed as

(38) y(t)=ℜ(σ,τ,φ,θ)β+N(t),

where

(39) $$ℜ\left(\sigma ,\tau ,\varphi ,\theta \right)=\left[ℜ\left(\sigma_{1},\tau_{1},\varphi_{1},\theta_{1}\right),\ldots ,ℜ\left(\sigma_{L},\tau_{L},\varphi_{L},\theta_{L}\right)\right],$$

(40) $$ℜ\left(\sigma_{l},\tau_{l},\varphi_{l},\theta_{l}\right)=\left[x\left(\sigma_{l},\tau_{l},t\right)\circ B\left(\varphi_{l}\right)\right]⊙A\left(\theta_{l}\right),$$

and $\beta ≜{\left[\beta_{1},\ldots ,\beta_{L}\right]}^{T}$, $x\left(\sigma ,\tau ,t\right)=\left[x\left(\left(t-\tau_{1}\right)/\sigma_{1}\right),x\left(\left(t-\tau_{2}\right)/\sigma_{2}\right),\ldots ,x\left(\left(t-\tau_{L}\right)/\sigma_{L}\right)\right]$.

The four parameters to be estimated are the time delay $\tau ≜\left[\tau_{1},\tau_{2},\ldots ,\tau_{L}\right]$, the Doppler stretch $\sigma ≜\left[\sigma_{1},\sigma_{2},\ldots ,\sigma_{L}\right]$, the DOD $\varphi =\left[\varphi_{1},\varphi_{2},\ldots ,\varphi_{L}\right]$, and the DOA $\theta =\left[\theta_{1},\theta_{2},\ldots ,\theta_{L}\right]$, which form the parameter vector ξ as $\xi ={\left[a,\tau ,\varphi ,\theta \right]}^{T}$.

The element at i,j in the Fisher information matrix (FIM) for estimating the vector ξ can be depicted as follows [21,32,36,37,38]:

(41) Γij(ξ)=2Re∑t=1N{(∂ℜ(σ,τ,φ,θ)β∂ξi)HQn−1(∂ℜ(σ,τ,φ,θ)β∂ξj)},

where N denotes the number of snapshots, and $Q_{n}=S_{0}I_{N}$.

For Equation (41), we may calculate the partial derivations.

(42) $$\begin{array}{ll} \frac{\partial \left\{\beta^{H}{ℜ}^{H}\left(\sigma ,\tau ,\varphi ,\theta \right)\right\}}{\partial \sigma_{l}} & =\beta^{H}A^{H}\left(\theta \right)e_{l}e_{l}^{T}⊙{\left[{x^{\prime }}_{\left(\sigma \right)}\left(\sigma ,\tau ,t\right)\circ B\left(\varphi \right)\right]}^{H}\\ & =\beta^{H}e_{l}e_{l}^{T}A^{H}\left(\theta \right)⊙{\left[{x^{\prime }}_{\left(\sigma \right)}\left(a,\tau ,t\right)\circ B\left(\varphi \right)\right]}^{H}\\ & =\beta^{H}e_{l}e_{l}^{T}{{ℜ}^{\prime }}_{a}^{H}\left(\sigma ,\tau ,\varphi ,\theta \right) \end{array},$$

where $e_{l}$ is the l-th column of the identity matrix (i.e., vector containing one in the l-th position and zeroes elsewhere). The following notations are introduced:

(43) $${{ℜ}^{\prime }}_{\sigma }\left(\sigma ,\tau ,\varphi ,\theta \right)=\frac{\partial ℜ\left(\sigma ,\tau ,\varphi ,\theta \right)}{\partial \sigma }=\left[{x^{\prime }}_{\left(\sigma \right)}\left(\sigma ,\tau ,t\right)\circ B\left(\varphi \right)\right]⊙A\left(\theta \right),$$

where ∘ is the Schur–Hadamard matrix product and ⊙ is Khatri–Rao matrix product.

(44) $${x^{\prime }}_{\left(\sigma \right)}\left(\sigma ,\tau ,t\right)=\left[\frac{\partial x\left(\left(t-\tau_{1}\right)/\sigma_{1}\right)}{\partial \sigma_{1}},\frac{\partial x\left(\left(t-\tau_{2}\right)/\sigma_{2}\right)}{\partial \sigma_{2}},\ldots ,\frac{\partial x\left(\left(t-\tau_{L}\right)/\sigma_{L}\right)}{\partial \sigma_{L}}\right].$$

From Equations (42)–(44), we obtain that

(45) $$\begin{array}{ll} \frac{\partial {\left\{ℜ\left(\sigma ,\tau ,\varphi ,\theta \right)\beta \right\}}^{H}}{\partial \sigma } & =\frac{\partial \left\{\beta^{H}{ℜ}^{H}\left(\sigma ,\tau ,\varphi ,\theta \right)\right\}}{\partial \sigma }\\ & ={\left[{{ℜ}^{\prime }}_{a}\left(\sigma ,\tau ,\varphi ,\theta \right)e_{1}e_{1}^{T}\beta ,\ldots ,{{ℜ}^{\prime }}_{a}\left(\sigma ,\tau ,\varphi ,\theta \right)e_{L}e_{L}^{T}\beta \right]}^{H}\\ & =\Delta^{H}{\left({{ℜ}^{\prime }}_{\alpha }\left(\sigma ,\tau ,\varphi ,\theta \right)\right)}^{H} \end{array},$$

where $\Delta =diag\left\{\beta_{1},\beta_{2},\ldots ,\beta_{L}\right\}$.

(46) Γσσ(ξ)=2Re∑t=1N{(∂ℜ(σ,τ,φ,θ)β∂σ)HQn−1(∂ℜ(σ,τ,φ,θ)β∂σ)}=2Re∑t=1N{∂{βHℜH(σ,τ,φ,θ)}∂σQn−1∂{ℜ(σ,τ,φ,θ)β}∂σ}=2Re∑t=1N{ΔH(ℜ′σ(σ,τ,φ,θ))HQn−1(ℜ′σ(σ,τ,φ,θ))Δ}.

Using Equation (46), the following explicit expressions for the blocks of the FIM were derived for the proposed signal model:

(47) Γττ(ξ)=2Re∑t=1N{ΔH(ℜ′τ(σ,τ,φ,θ))HQn−1(ℜ′τ(σ,τ,φ,θ))Δ};

(48) Γφφ(ξ)=2Re∑t=1N{ΔH(ℜ′φ(σ,τ,φ,θ))HQn−1(ℜ′σ(σ,τ,φ,θ))Δ};

(49) Γθθ(ξ)=2Re∑t=1N{ΔH(ℜ′θ(σ,τ,φ,θ))HQn−1(ℜ′θ(σ,τ,φ,θ))Δ};

(50) Γστ(ξ)=2Re∑t=1N{ΔH(ℜ′σ(σ,τ,φ,θ))HQn−1(ℜ′τ(σ,τ,φ,θ))Δ};

(51) Γaφ(ξ)=2Re∑t=1N{ΔH(ℜ′σ(σ,τ,φ,θ))HQn−1(ℜ′φ(σ,τ,φ,θ))Δ};

(52) Γaθ(ξ)=2Re∑t=1N{ΔH(ℜ′σ(σ,τ,φ,θ))HQn−1(ℜ′θ(σ,τ,φ,θ))Δ};

(53) Γτφ(ξ)=2Re∑t=1N{ΔH(ℜ′τ(σ,τ,φ,θ))HQn−1(ℜ′φ(σ,τ,φ,θ))Δ};

(54) Γτθ(ξ)=2Re∑t=1N{ΔH(ℜ′τ(σ,τ,φ,θ))HQn−1(ℜ′θ(σ,τ,φ,θ))Δ};

(55) Γφθ(ξ)=2Re∑t=1N{ΔH(ℜ′φ(σ,τ,φ,θ))HQn−1(ℜ′θ(σ,τ,φ,θ))Δ};

where

(56) $$\Delta =diag\left\{\beta_{1},\beta_{2},\ldots ,\beta_{L}\right\};$$

(57) $${{ℜ}^{\prime }}_{\sigma }\left(\sigma ,\tau ,\varphi ,\theta \right)=\frac{\partial {ℜ}_{\tau }\left(\sigma ,\tau ,\varphi ,\theta \right)}{\partial \sigma }=\left[{x^{\prime }}_{\left(\sigma \right)}\left(\sigma ,\tau ,t\right)\circ B\left(\varphi \right)\right]⊙A\left(\theta \right);$$

(58) $${x^{\prime }}_{\left(\sigma \right)}\left(\sigma ,\tau ,t\right)≜\left[\frac{\partial x\left(\left(t-\tau_{1}\right)/\sigma_{1}\right)}{\partial \sigma_{1}},\frac{\partial x\left(\left(t-\tau_{2}\right)/\sigma_{2}\right)}{\partial \sigma_{2}},\ldots ,\frac{\partial x\left(\left(t-\tau_{L}\right)/\sigma_{L}\right)}{\partial \sigma_{L}}\right];$$

(59) $${{ℜ}^{\prime }}_{\tau }\left(\sigma ,\tau ,\varphi ,\theta \right)=\left[{x^{\prime }}_{\left(\tau \right)}\left(\sigma ,\tau ,t\right)\circ B\left(\varphi \right)\right]⊙A\left(\theta \right);$$

(60) $${x^{\prime }}_{\left(\tau \right)}\left(\sigma ,\tau ,t\right)≜\left[\frac{\partial x\left(\left(t-\tau_{1}\right)/\sigma_{1}\right)}{\partial \tau_{1}},\frac{\partial x\left(\left(t-\tau_{2}\right)/\sigma_{2}\right)}{\partial \tau_{2}},\ldots ,\frac{\partial x\left(\left(t-\tau_{L}\right)/\sigma_{L}\right)}{\partial \tau_{L}}\right];$$

(61) $${{ℜ}^{\prime }}_{\varphi }\left(\sigma ,\tau ,\varphi ,\theta \right)=\left[x\left(\sigma ,\tau ,t\right)\circ B^{\prime }\left(\varphi \right)\right]⊙A\left(\theta \right);$$

(62) $$B^{\prime }\left(\varphi \right)=\left[\frac{\partial B\left(\varphi_{1}\right)}{\partial \varphi_{1}},\frac{\partial B\left(\varphi_{2}\right)}{\partial \varphi_{2}},\ldots ,\frac{\partial B\left(\varphi_{L}\right)}{\partial \varphi_{L}}\right];$$

(63) $${{ℜ}^{\prime }}_{\theta }\left(\sigma ,\tau ,\varphi ,\theta \right)=\left[x\left(\sigma ,\tau ,t\right)\circ B\left(\varphi \right)\right]⊙A^{\prime }\left(\theta \right);$$

(64) $$A^{\prime }\left(\theta \right)=\left[\frac{\partial A\left(\theta_{1}\right)}{\partial \theta_{1}},\frac{\partial A\left(\theta_{2}\right)}{\partial \theta_{2}},\ldots ,\frac{\partial A\left(\theta_{L}\right)}{\partial \theta_{L}}\right].$$

The expression for the CRB, shown in Equation (65), is obtained by substituting Equations (46)–(64) into Equation (39).

(65) $$\mathrm{CRB}\left(\xi \right)=\Gamma^{-1}.$$

### 5.4. Complexity Analysis

In this section, we evaluate the computation complexity of the proposed method.

#### 5.4.1. Doppler Stretch and Time Delay

The method based on the WBAF can estimate Doppler stretch and time delay by searching the peak of the WBAF. Denoting the number of snapshots, time delay, and Doppler stretch to be searched as $N_{s}$, $N_{\tau }$, and $N_{\sigma }$, respectively, the computational complexity of the WBAF is then around $O\left(N_{\tau }N_{\sigma }N_{s}\right)$ [39,40]. The computation complexity of the FLOS-WBAF algorithm is $O\left(N_{\tau }N_{\sigma }N_{s}\right)$. The parameter estimation based on Sigmoid-WBAF method is a two-step process. The first step carries the Sigmoid transformation and the second step computes the WBAF. Accordingly, the computational complexity of the Sigmoid-WBAF algorithm is $O\left(N_{\tau }N_{\sigma }N_{s}+N_{s}\right)\approx O\left(N_{\tau }N_{\sigma }N_{s}\right)$. Through the computational complexity analysis, the Sigmoid-WBAF method not only has the same computation complexity as the WBAF method and FLOS-WBAF method, but also can suppress impulsive noise interference and does not need any a priori knowledge of the noise.

#### 5.4.2. DOD and DOA

The computational complexities of the proposed Sigmoid-MUSIC, MUSIC, and FLOM-MUSIC algorithms were compared. All these methods include the eigen decomposition step which is represented by the term $O\left(N^{3}\right)$. The computation of J samples of the MUSIC spectrum function requires O(JNL). Thus, the computational complexity of spectral MUSIC is $O\left(N^{3}+JNL\right)$, where N denotes the number of the received antennas, and L denotes the number of the targets [41]. The computational complexity of FLOM-MUSIC is also $O\left(N^{3}+JNL\right)$. The Sigmoid-MUSIC method needs to carry the Sigmoid transformation in advance. Thus, the computational complexity of Sigmoid-MUSIC is $O\left(N^{3}+JNL+N_{s}\right)\approx O\left(N^{3}+JNL\right)$.

Through the computational complexity analysis, we can deduce that the Sigmoid-MUSIC method not only has the same computation complexity as other methods, but also can suppress impulsive noise interference and does not need any a priori knowledge of the noise.

## 6. Simulation Results

We performed simulation experiments to assess the relative performance of the different methods, including the WBAF [12], FLOS-WBAF [18], and Sigmoid-WBAF to estimate TD and DS; and the conditional MUSIC [21], FLOM-MUSIC [22], FLOM-SC-SSF [14], $l_{p}$-MUSIC [24], and Sigmoid-MUSIC to estimate DOD and DOA, under α stable noise condition.

The wideband bistatic MIMO radar was composed of Q=6 transmit antennas and N=8 receive antennas. Suppose that the target is located at the positions $\left(\varphi_{1},\theta_{1}\right)=\left(10^{o},50^{o}\right)$, $\left(\varphi_{2},\theta_{2}\right)=\left(40^{\circ },30^{\circ }\right)$; DS and TD are $\sigma_{1}=1.2$, $\tau_{1}=30/f_{s}$, $\sigma_{2}=0.9$, $\tau_{2}=10/f_{s}$, respectively, where the sampling frequency $f_{s}$ is 1KHz. The root-mean-square error (RMSE) is defined as

(66) $$\mathrm{RMSE}=\frac{1}{2}\left(\sqrt{\frac{1}{K}{\sum_{k=1}^{K}\left[{\hat{x}}_{1}\left(k\right)-x_{1}\right]}^{2}}+\sqrt{\frac{1}{K}{\sum_{k=1}^{K}\left[{\hat{x}}_{2}\left(k\right)-x_{2}\right]}^{2}}\right),$$

where ${\hat{x}}_{1}$ and ${\hat{x}}_{2}$ are the estimations of $x_{1}$ and $x_{2}$, and K is the total number of successful runs. For each simulation, the numbers of Monte Carlo runs and snapshots were 300 and 1000, respectively.

### 6.1. Simulation 1: Spectra of WBAF, FLOS-WBAF, and Sigmoid-WBAF for a Single Estimation for Two Targets

**Figure 3:**
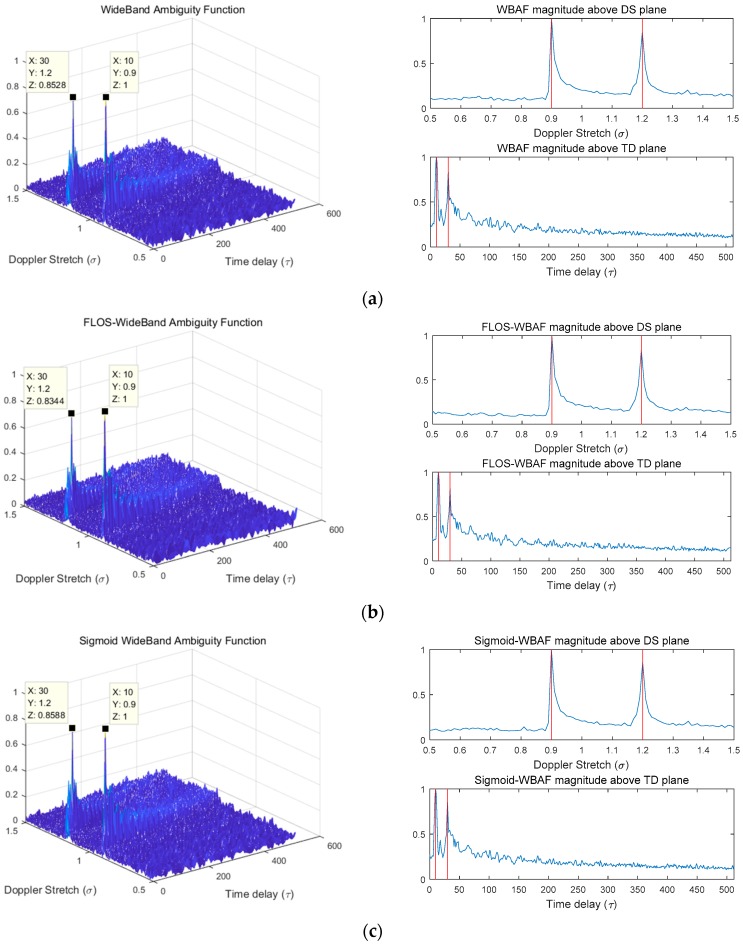
The normalized spectra of WBAF, FLOS-WBAF, and Sigmoid-WBAF for Gaussian noise with SNR=−10 dB. (**a**) The three-dimensional (3D) plot of WBAF and its Doppler stretch (DS) and time delay (TD) section planes; (**b**) the 3D plot of FLOS-WBAF with *p* = 1.1 and its DS and TD section planes; (**c**) the 3-D plot of Sigmoid-WBAF and its DS and TD section planes.

**Figure 4:**
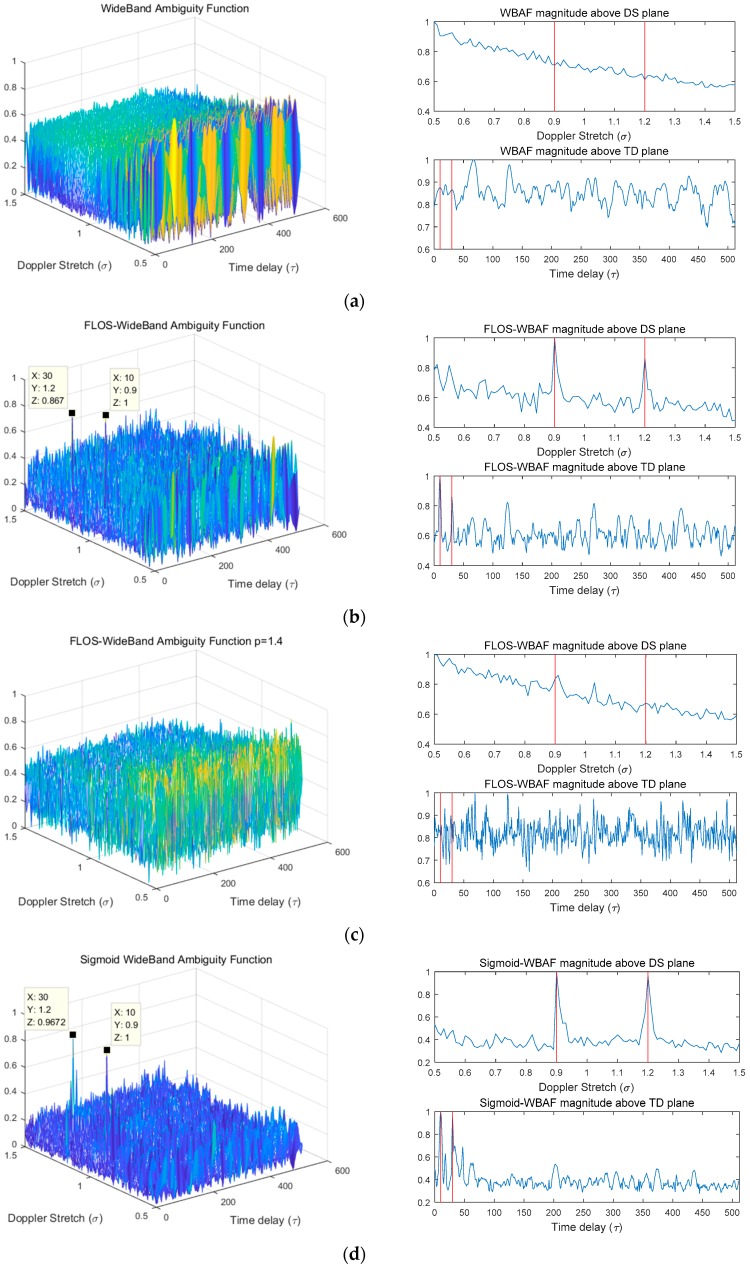
The normalized spectra of WBAF, FLOS-WBAF, and Sigmoid-WBAF for impulsive noise with GSNR=2 dB and α=1.2. (**a**) The 3D plot of WBAF and its DS and TD section planes; (**b**) the 3D plot of FLOS-WBAF with *p* = 1.1 and its DS and TD section planes; (**c**) the 3D plot of FLOS-WBAF with *p* = 1.4 and its DS and TD section planes; (**d**) the 3D plot of Sigmoid-WBAF and its DS and TD section planes.

**Figure 5:**
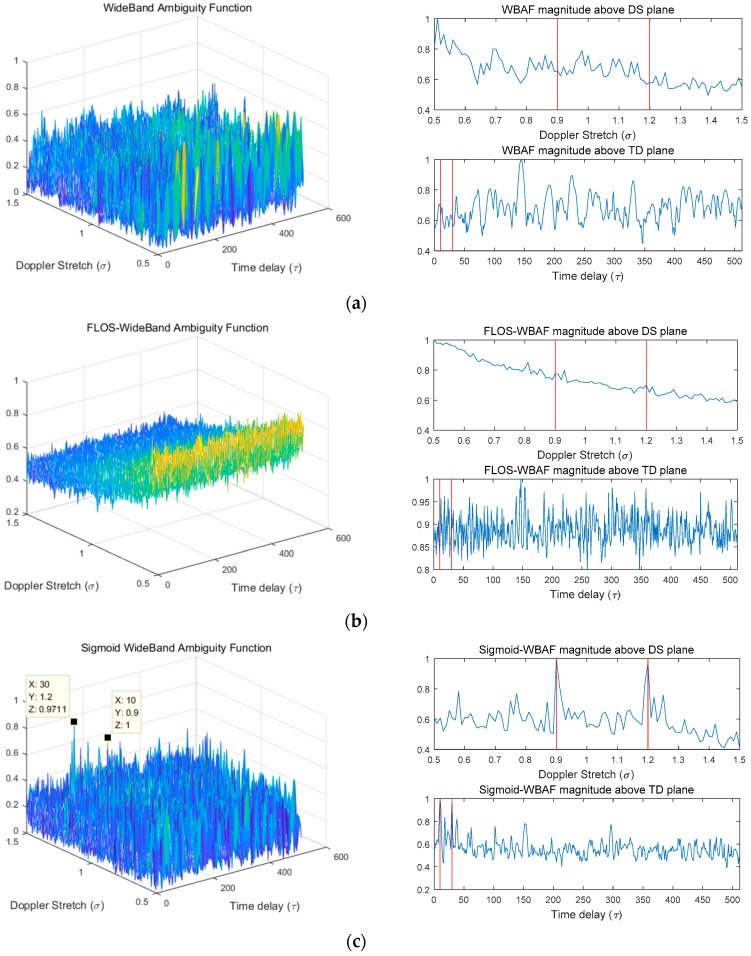
The normalized spectra of WBAF, FLOS-WBAF, and Sigmoid-WBAF for impulsive noise with GSNR=−2 dB and α=1.2. (**a**) The 3D plot of WBAF and its DS and TD section planes; (**b**) the 3D plot of FLOS-WBAF with *p* = 1.1 and its DS and TD section planes; (**c**) the 3D plot of Sigmoid-WBAF and its DS and TD section planes.

In this simulation, we discuss the estimation results obtained from the WBAF, FLOS-WBAF, and Sigmoid-WBAF for two targets in the Gaussian noise and impulsive noise environment. The corresponding results are illustrated in Figure 3, Figure 4 and Figure 5.

In the Gaussian noise, all three methods revealed two clear peaks, as illustrated in Figure 3. However, from Figure 4 and Figure 5, it can clearly be seen that the WBAF algorithm failed for the impulsive noise case. The reason is that the WBAF method does not have the ability to suppress impulsive noise. On the other hand, the FLOS-WBAF algorithm, combining the fractional lower-order statistics theory with the wideband ambiguity function, can effectively suppress the alpha stable noise interference, where clear peaks were obtained for the impulsive noise case with GSNR=2 dB, α=1.2, and p=1.1. However, FLOS-WBAF failed to obtain the correct spectrum peaks for impulsive noise with α=1.2 and p=1.4, mainly due to the fact that the fractional lower-order moment p value was not appropriate, as illustrated in Figure 4. Based on the fractional lower-order statistics theory, the characteristic exponent of the noise must be estimated to ensure 1≤p<α or 0<p<α/2. The methods employing the FLOS theory cannot accurately estimate the parameters if there is no a priori knowledge of the characteristic exponent. Furthermore, the algorithm’s performance degrades seriously and even becomes invalid when the fractional lower-order moment value is outside the specified range. On the contrary, Sigmoid-WBAF had clear peaks regardless of the value of the fractional lower-order moment p. As illustrated in Figure 5, FLOS-WBAF failed to obtain the correct peaks for impulsive noise with GSNR=−2dB, α=1.2, and p=1.1. However, Sigmoid-WBAF had clear peaks. The reason is that the Sigmoid transform can suppress impulsive noise better than any of the FLOS-type methods, including FLOS-WBAF.

### 6.2. Simulation 2: Spectrum Performances of the Four Algorithms

In this simulation, the generalized signal-to-noise ratio was set to GSNR = 0 dB and the characteristic exponent α was set to 1.2.

**Figure 6:**
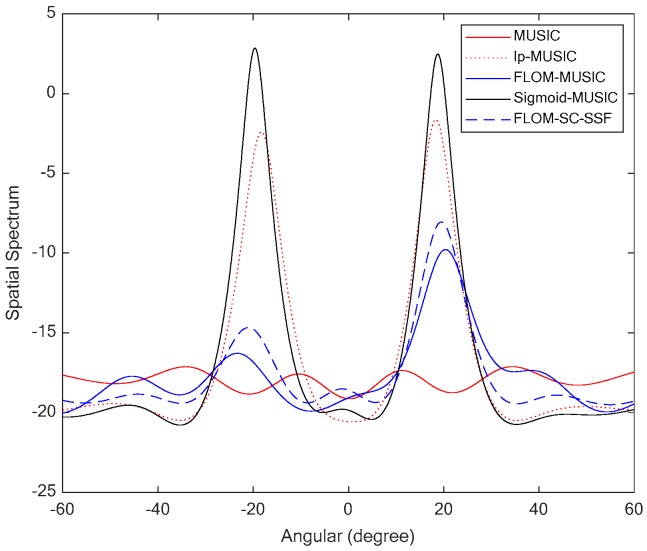
Spatial spectra of the four algorithms.

Figure 6 shows the spatial spectra of the MUSIC, FLOM-MUSIC, $l_{p}$-MUSIC, FLOM-SC-SSF, and Sigmoid-MUSIC algorithms. The performance of the MUSIC algorithm degraded seriously under the α stable distribution noise. Although the spatial spectra of the FLOM-MUSIC, FLOM-SC-SSF, and $l_{p}$-MUSIC algorithms had two spectrum peaks, the spectrum peak of FLOM-MUSIC showed a deviation from the true location, and the spectrum peaks of FLOM-SC-SSF and $l_{p}$-MUSIC did not have two spiked peaks. The Sigmoid-MUSIC algorithm based on Sigmoid transform can suppress the impulse noise interference, especially for low GSNR. Therefore, the Sigmoid-MUSIC algorithm had a more accurate spectrum peak and better estimation performance.

### 6.3. Simulation 3: Generalized Signal-to-Noise Ratio (GSNR)

**Figure 7:**
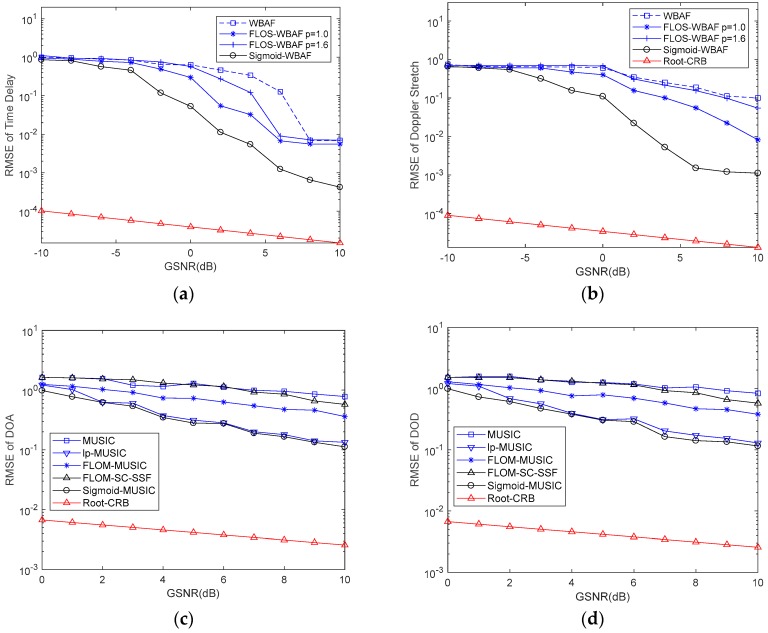
Root-mean-square errors (RMSEs) of parameters versus generalized signal-to-noise ratio (GSNR). (**a**) RMSE of the time delay; (**b**) RMSE of the Doppler stretch; (**c**) RMSE of the direction-of-departure (DOD); (**d**) RMSE of the direction-of-arrival (DOA).

In this simulation and to evaluate the performances of TD and DS, the characteristic exponent α was set to α=1.2, and the fractional lower-order moment p was set to p=1.0 and p=1.6 for FLOS-WBAF. The parameter *p* was set to p=1.4 for FLOM-MUSIC while estimating the performances of DOD and DOA. The root CRBs and RMSEs of parameter estimation under different GSNRs are shown in Figure 7.

From Figure 7a,b, we can find that the WBAF method had poor estimation performance. The estimation performance of FLOS-WBAF was affected by the fractional-lower order moment p. The estimation performance of FLOS-WBAF with p>α had poor performance. The RMSE of Sigmoid-WBAF was obviously lower than that of FLOS-WBAF and WBAF when GSNR<5. When GSNR≥5, the performance of Sigmoid-WBAF was similar to the other methods. From Figure 7c,d, the conventional MUSIC method was inferior to the other methods. For low GSNRs, both Sigmoid-MUSIC and $l_{p}$-MUSIC could get lower RMSEs than other methods; however, Sigmoid-MUSIC yielded more accurate DOA estimation results. Therefore, the estimation performance of the proposed method was superior to other methods. As shown in Figure 7, the root CRB decreased with increasing GSNR. Moreover, the root CRB could be apparently affected by the GSNR for a given characteristic exponent α.

### 6.4. Simulation 4: Characteristic Exponent α

**Figure 8:**
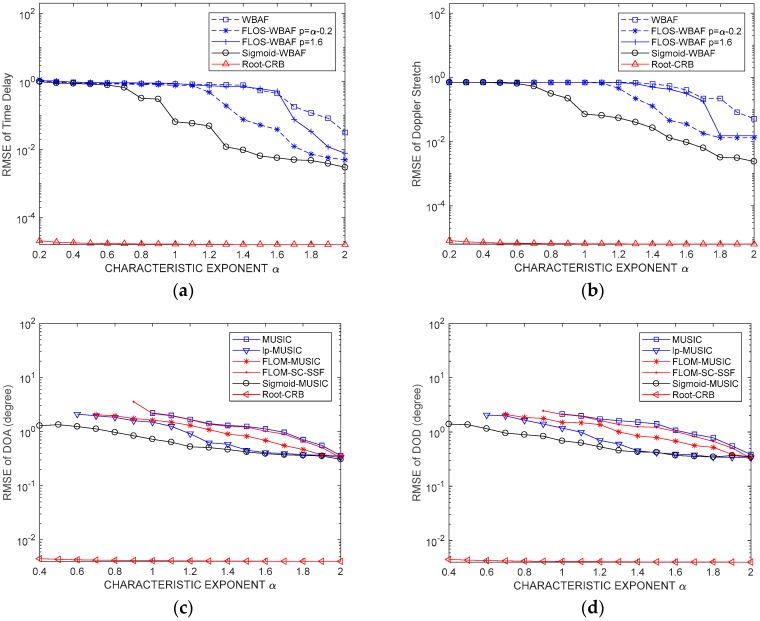
RMSEs of the parameters versus characteristic exponent α. (**a**) RMSE of the time delay; (**b**) RMSE of the Doppler stretch; (**c**) RMSE of the DOD; (**d**) RMSE of the DOA.

In this simulation and to measure the estimation performances of TD and DS, the fractional lower-order moment p was set to p=α−0.2 and p=1.4 for FLOS-WBAF. The GSNR was set to GSNR=5dB. While evaluating the performances of DOD and DOA, the fractional lower-order moment p was set to p=1.4 for FLOM-MUSIC. The root CRBs and RMSEs on parameter estimation under different characteristic exponents α are shown in Figure 8.

From Figure 8a,b, we can find that WBAF and FLOS-WBAF with p=1.6 had poor estimation performance. When 0.5<α<1.5, the estimation performance of Sigmoid-WBAF was obviously better than that of the FLOS-WBAF method with p<α. Although FLOS and Sigmoid transform can both suppress impulsive noise, the suppression capacity of FLOS was not enough because ${\left|x_{2}\left(t\right)\right|}^{p}>{\left|x_{1}\left(t\right)\right|}^{p}>1$ for any $\left|x_{2}\left(t\right)\right|>\left|x_{1}\left(t\right)\right|>1$. For this reason, large outliers of impulsive noise cannot be restrained sufficiently when the impulsiveness is extremely intensive. In contrast, the Sigmoid function can be assumed to be approximately linear. |x(t)| can reach significant suppression when |x(t)| is far away from zero. Because the signals are often assumed to have a zero-mean value, the Sigmoid function can be used to suppress the outliers [42]. For any |x(t)|>1, ${\left|x_{2}\left(t\right)\right|}^{p}>{\left|x_{1}\left(t\right)\right|}^{p}>1>\left|\mathrm{Sigmoid}\left[x\left(t\right)\right]\right|$. Therefore, the Sigmoid function suppresses the outliers much better than FLOS, and the estimation performance of the Sigmoid-WBAF algorithm was better than that of the FLOS-WBAF algorithm. From Figure 8c,d, we can find that the conventional MUSIC method was inferior to other methods. For highly impulsive noise, Sigmoid-MUSIC could get more accurate DOA estimation results. When α≥1, Sigmoid-MUSIC and $l_{p}$-MUSIC could obtain smaller RMSEs than other methods. When α=2, the α stable distribution became a Gaussian distribution. As shown in Figure 8, in this case, the three algorithms had similar performance. As illustrated in Figure 8, root CRB decreased with increasing characteristic exponent α; however, root CRB was less affected by α for a given GSNR.

## 7. Conclusions

In this paper, we considered the problem of parameter estimation for a wideband bistatic MIMO radar in impulsive noise environments. Based on the WBAF and MUSIC methods, we developed a novel method without any a priori knowledge of the noise. A robust estimator, termed Sigmoid-WBAF, was employed to estimate the time delay and Doppler stretch in the presence of impulsive noise. Then, the properties of the Sigmoid transform and Sigmoid correlation were presented. A novel MUSIC-based Sigmoid correlation (Sigmoid-MUSIC) was developed to estimate DOD and DOA. Furthermore, the boundness of Sigmoid-WBAF to the symmetric alpha stable (SαS) noise, the feasibility analysis of Sigmoid-WBAF, and complexity analysis of Sigmoid-WBAF and Sigmoid-MUSIC algorithms were presented to evaluate the performance of the proposed method. In addition, the Cramér–Rao bound for parameter estimation was derived and computed in closed form, which showed that better performance was achieved. Lastly, comprehensive simulations were carried out to evaluate the performance of different methods. Simulation results and theoretical analyses showed that the proposed Sigmoid-WBAF and Sigmoid-MUSIC had better estimation performance, especially in highly impulsive noise environments.
